# Bibliometric analysis of research trends of physical activity intervention for autism spectrum disorders

**DOI:** 10.3389/fnhum.2022.926346

**Published:** 2022-08-12

**Authors:** Shimeng Wang, Dandan Chen, Inae Yoon, Sebastian Klich, Aiguo Chen

**Affiliations:** ^1^College of Physical Education, Yangzhou University, Yangzhou, China; ^2^Institute of Sports, Exercise and Brain, Yangzhou University, Yangzhou, China; ^3^Chinese–Polish Laboratory of Sport and Brain Science, Yangzhou University, Yangzhou, China; ^4^Graduate School of Education (Department of Pedagogy: Physical Education), Dankook University, Yongin-si, South Korea; ^5^Department of Paralympic Sport, Wroclaw University of Health and Sport Sciences, Wrocław, Poland

**Keywords:** bibliometric analysis, research trend, physical activity, autism spectrum disorders, CiteSpace, hotspots and frontiers

## Abstract

Autism spectrum disorder (ASD) is a neurodevelopmental disorder characterized by social impairment, restricted interests, and repetitive stereotyped behaviors. At present, its pathogenesis has not been fully understood. Various methods are used for clinical treatment and intervention, among which physical activity (PA) intervention also has an obvious effect. This study has used bibliometric methods and visual analysis methods to analyze 885 studies of PA intervention in ASD from 2003 to 2022 in the Web of Science (WoS) database in order to provide theoretical support for the follow-up research on the effect of PA with ASD. The main findings of this study are as follows. First, the literature on PA interventions in ASD research showed a growing trend. The leading institution in this field is the University of Delaware, forming a core group of authors represented by authors such as Sean Healy and Carol Curtin et al. Second, the research focus of this research area mainly includes PA interventions for children and adolescents with ASD. PA can improve symptoms such as stereotyped behaviors and motor function in patients with ASD as well as can reduce childhood obesity rates and improve quality of life. Third, skill, youth, prevalence, and meta-analysis systematic reviews were found. It is the long-term concern and focus of researchers. In conclusion, the current research is only a short-term analysis, and it is not possible to verify the long-term effect; thus, future data analysis should evaluate and explore the long-term effects of PA interventions on ASD including cohort and longitudinal study types focused on the rehabilitation of patients with ASD. Moreover, testing the sustainability of benefits for children with ASD and constructing a multidimensional exercise integrated intervention model are the main directions for future research in this field.

## Introduction

Autism spectrum disorder (ASD) is a lifelong neurodevelopmental disorder that limits or impairs daily functioning characterized by social communication, social interaction disorders, narrow interests, and repetitive stereotypes (Association American Psychiatric, 2013). The prevalence of ASD is increasing every year, while it usually has a high lifetime disability rate (Hodges et al., 2020). According to the Centers for Disease Control and Prevention, the prevalence of ASD has increased from 2 per 10,000 children to 1 per 54 children over the past 20 years (Maenner et al., 2020). For more than half a century, multidisciplinary research has been devoted to exploring the causes and pathogenesis of ASD, but, so far, it has not been explained, so there is no definitive curative treatment.

Studies in neuroscience have linked physical activity (PA) to brain structure and cognitive development in recent years (Donnelly et al., 2016). Previous systematic reviews and meta-analyses have shown a positive effect of PA interventions on cognitive function, especially on working memory, selective attention inhibition, and cognitive flexibility (Álvarez-Bueno et al., 2017). The positive effect on cognitive function is related to an increase of brain-derived neurotrophic factors, thereby promoting learning and maintaining cognitive function by improving synaptic plasticity and electrical stimulation of a nerve and increasing cerebral circulation (Hillman et al., 2008). Moreover, the PA has an important effect on cognitive function in patients with ASD. Pan et al. (2017) significantly reported higher motor skill proficiency and executive function after 12 weeks of PA intervention in children with ASD. Moreover, a large number of experimental studies have also found that PA interventions can improve social interaction disorders, stereotyped behaviors (Teh et al., 2021), language disorders (Zeng et al., 2017), athletic ability (Cai et al., 2020; Park et al., 2021), behavioral management (Greco and De Ronzi, 2020), and lowering body fat (Sefen et al., 2020). PA is increasingly valued as an intervention method without adverse reactions. It promotes an increase in the review studies of PA intervention in ASD.

As a medium for documenting research results, a review is an important source of theoretical results. Through the review, the research trends and their development status can be clearly understood, laying the foundation for future scientific research. So far, scholars have published a number of different studies that investigated the effect of PA intervention in ASD, and how to extract information from many existing studies and clarifying the research status and development trend in this field is a valuable Research Topic. The bibliometric analysis uses quantitative methods to examine the structure and development of different disciplines and is a process of evaluating and predicting the current situation and development trends in the research field using measurement methods such as mathematics and statistics (Zupic and Cater, 2015; Van Raan, 2019). Through the analysis of institutions, authors, and keywords in bibliometric analysis (Yu et al., 2017), scholars are able to obtain the research status and trends of PA intervention in ASD research. Quantitative analysis of the literature to determine differences between studies in different regions and the merits of author collaboration (Ellegaard and Wallin, 2015) both circumvents the bias of researchers and has the advantage of identifying important research content (Pesta et al., 2018).

In recent years, many researchers have conducted a large number of bibliometric analysis studies on ASD by using bibliometric analysis. Reviewing the findings related to this study objective, Sweileh et al. (2016) conducted an author collaboration network analysis of global ASD studies using bibliometric methods, revealing the characteristics and trends of domain collaboration. Wang et al. (2020) found that *de novo* mutations (DNMs) are a hot topic in the field of ASD research in recent years and believe that genetic information is an important research trend in the field of ASD research. Shekarro et al. (2021) analyzed the institutional and author partnerships, citations, and funding funds of ASD executive function research, providing a reference for subsequent authors to understand the knowledge structure and development trend of ASD executive function research.

In summary, bibliometrics has been successfully applied in the research field of ASD, but most of the existing studies are more comprehensive bibliometric analyses in the field of ASD research, and the specific research results of PA intervention in ASD research have not yet appeared. Therefore, it is necessary to summarize the latest progress and research direction and hotspot of PA intervention in ASD. In this case, a thorough understanding of PA intervention in the ASD research process is very important.

Therefore, the main aim of this study was to investigate both bibliometric and visual analysis methods to systematically analyze all the processes and reviews of PA interventions in ASD research included in the Web of Science (WoS) database. Understanding the structure, status, and future direction of the current PA intervention in ASD research will stimulate scholars to discover new problems and lay a further foundation for exploring intervention methods.

## Methods

### Data selected

The data in this study were derived from the core collection of WoS, an interdisciplinary comprehensive academic information database of the American Institute for Scientific Information (ISI), which were collected and screened through the WoS database. Based on the relevant prior studies (Healy et al., 2018), the search formula for this study was TS = (Autism spectrum disorder) AND TS = [physical activity (PA) intervention OR exercise rehabilitation OR exercise therapy OR exercise intervention OR physical activity OR exercise], file type was article or review, and the retrieval period was Searchable document start-2022.03.01 By reading the abstract and content of the article, the output literature data were manually screened and processed. Finally, 885 valid documents were obtained, and the search results of these documents were exported to the format required for CiteSpace operation, whose research data were used in this study.

### Data analysis methods

#### Bibliometric analysis methods

Bibliometric methods were quantitative and qualitative combinations of the number of authors, word frequency statistics, and citations in the literature (Zupic and Cater, 2015; Van Raan, 2019). In this study, publication time, institutions, authors, and keywords of studies were used to objectively evaluate the research status of PA intervention in ASD using the bibliometric analysis method.

#### Data visualization methods

**Figure 1:**
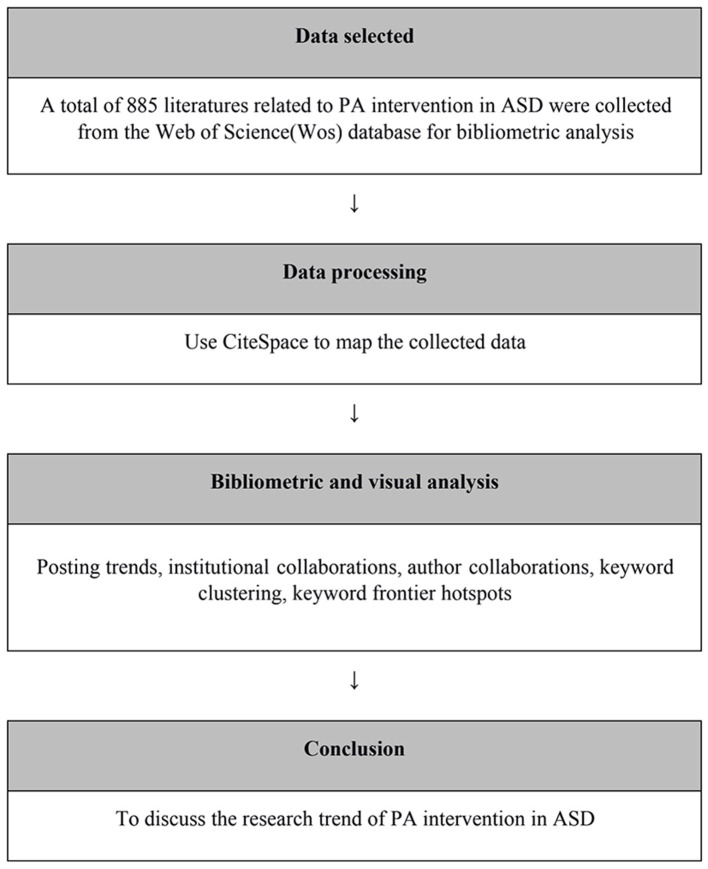
Process flow.

Data visualization methods were used to demonstrate graphical means to clearly and effectively convey and communicate information (Azzam et al., 2013). In this study, the CiteSpace (5.6.R1) (Chen, 2014) visual analysis software was used to visualize the development trend of PA intervention in ASD research. The CiteSpace is a visual analysis program developed by Professor Chaomei Chen of Drexel University based on JAVA, which has been widely used in visual analysis research in various fields of knowledge since its development. This study used CiteSpace to map the collected data in co-institutions' network analysis, co-author analysis, and keyword analysis. Thus, the research process and current situation of PA intervention in ASD research were explored, and the trend of future development was predicted. Based on the above, this research technology roadmap is shown in Figure 1.

## Result

### Basic statistical analysis

#### Published trend analysis

**Figure 2:**
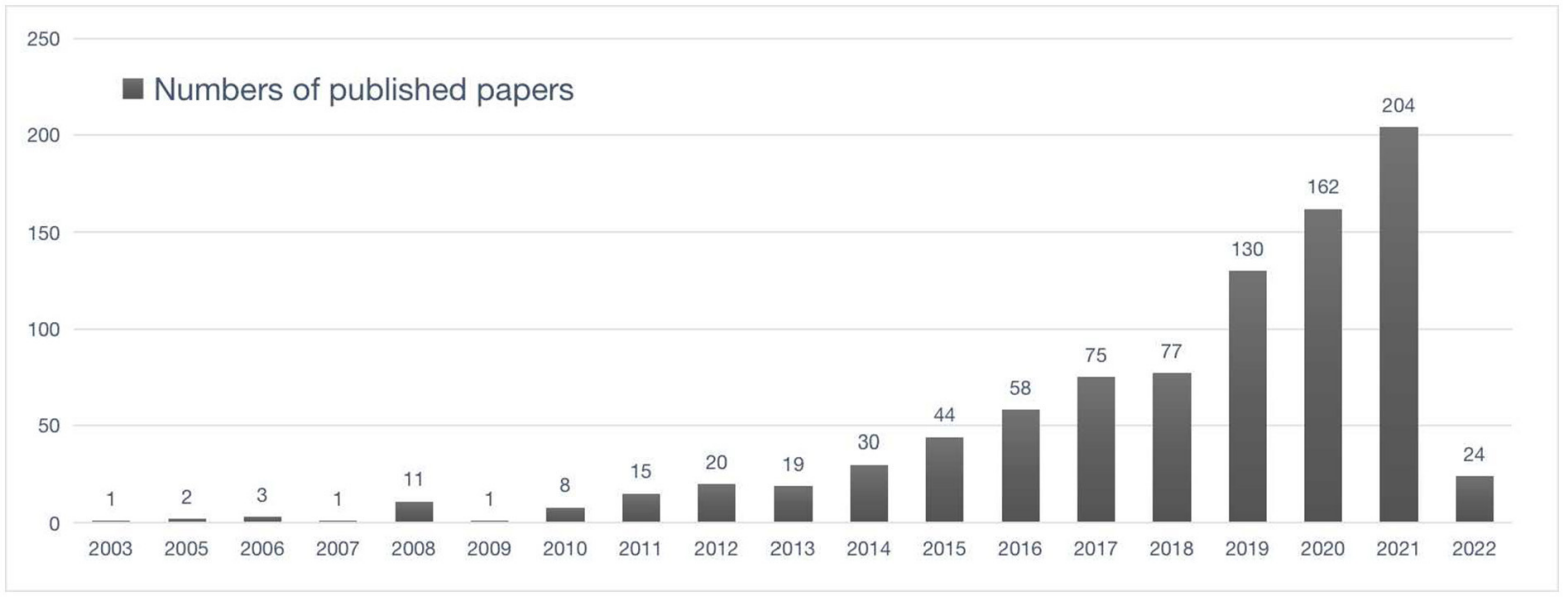
Publishing trend in the area of PA intervention for ASD (1 January 2003 to 1 March 2022).

The amount of writing may show the degree of attention of a certain subject area, and the continuous number of articles issued year by year may reflect the change in the degree of attention in the subject area. The number of literature published about ASD research from 1 January 2003, to 1 March 2022, showed a total of 885 articles. The number of articles published also showed a trend of increasing year by year (Figure 2), which was roughly divided into 3 stages. The first phase was the initial phase (2003–2013) from 1 article (2003) to 19 articles (2013), and a total of 81 articles were published during the 10 years, accounting for a total of 9.16%. The second phase was the slow growth phase (2014–2018), which increased from 44 articles (2014) to 130 articles (2018) during the 4 years, and a total of 284 articles were published, accounting for a total of 32.09%. The third phase was the high-speed phase (2019–2021), which increased from 130 articles (2019) to 204 articles (2021) during the 3 years, and a total of 496 articles were published, accounting for a total of 56.05%. During the period from 2003 to 2021, the literature on PA interventions in ASD research grew 200-fold. According to the publication trend, it is foreseeable that in the future, the number of relevant articles on PA intervention in ASD remains around 200–300 per annum. Note that the number of articles published in 2022 was 24 (2.7%) for the first 3 months of posting statistics.

The trend in publication volume shows that many researchers are gradually paying attention to the impact of PA on ASD. The reason for the increase in retrospective literature is largely related to the development and implementation of research measurement tools and research projects. In this study, although the rapid update of research measurement tools has made it difficult to track their specific time, the reason for the rapid increase in the number of studies since 2014 is closely related to brain programs implemented in countries around the world, such as the Human Brain Project (HBP), BRAIN Initiative, Brain/Minds Project, Brain Science and Brain-Like Intelligence Technology (China Brain Project), and other research projects oriented to tackling brain diseases. It has contributed to a significant increase in the amount, form, and latitude of data generated by new neuroscience instruments using imaging and activity recording, as well as an increased reliance on new technologies and analytical methods that utilize neuroscience data. For example, consider the HBP that aims to summarize the knowledge currently available to the human brain and gradually build models through supercomputers to simulate the human brain. The integration of neuroscience, medicine, and computer technology will enable humans to finally understand brain and brain diseases and offer new perspectives for future computers and robotics (https://www.humanbrainproject.eu/en/). These brain programs have not only promoted the innovation of imaging technology but also enabled humans to overcome unresolved brain diseases, indirectly increasing the number of publications in the field of PA intervention in ASD research.

#### Co-institutions' network

**Figure 3:**
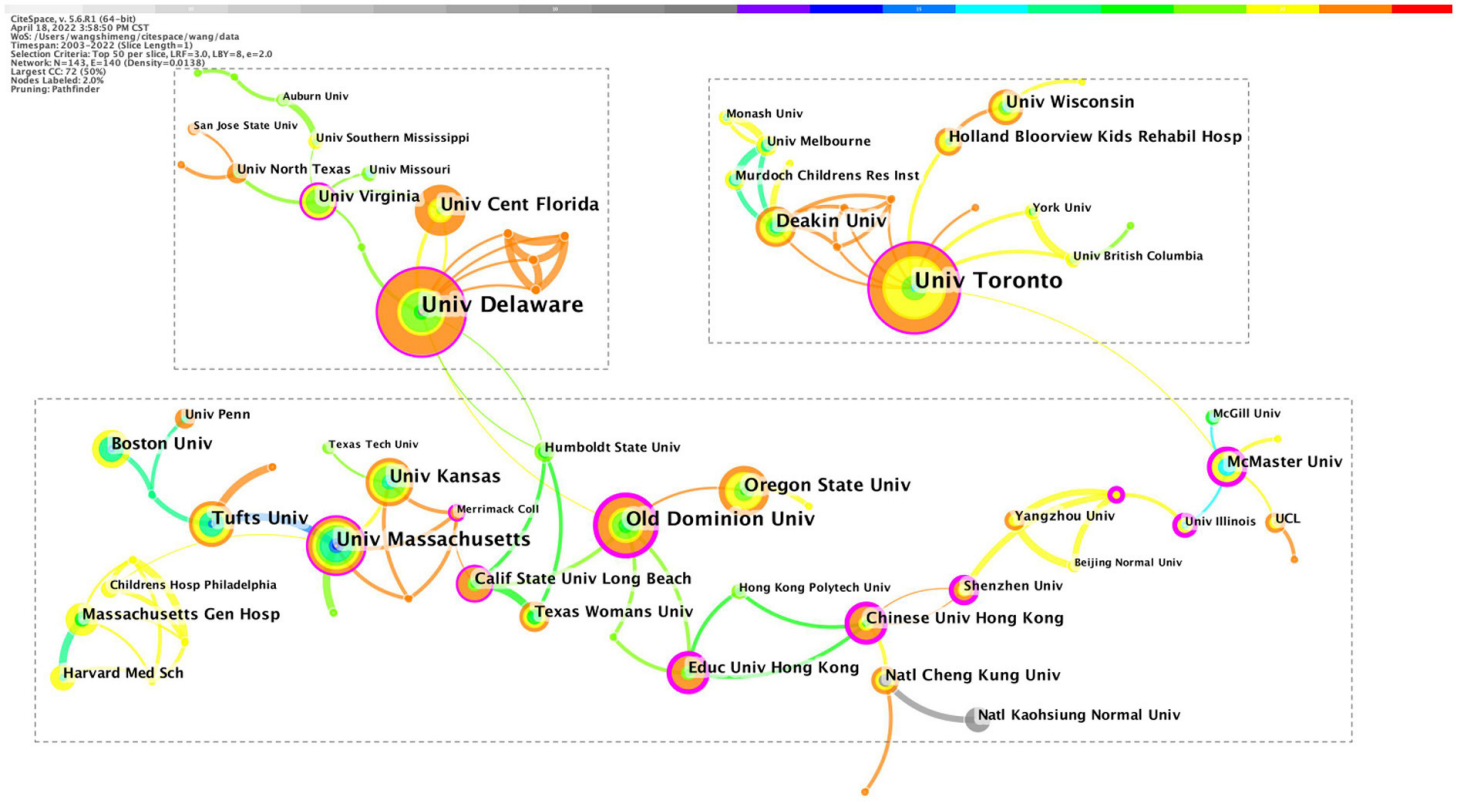
Co-institutions' network (2003–2022). The color of the circle represents when the article was published. The larger the node diameter, the more studies institutions have published. The thicker the line between the nodes, the closer the two institutions work together.

The use of CiteSpace generated a network as usual: 2003–2022; Slice length: 1 year; Select the node type: Institution; Top N = 50; and Choice: Pathfinder and Pruning the merged network. Other parameters were the default settings. In addition, the co-institutions' knowledge mapping was generated, in which N = 143 and E = 140 (density was 0.0138). Figure 3 and Table 1 indicate that the University of Delaware has published the most studies and has conducted strong scientific research in the study of PA intervention in ASD. The University of Delaware, University of Toronto, and Old Dominion University have formed 3 cooperation networks. Furthermore, the research institute formed a cooperative subnet with a size of 5 from 2015 to 2020. The Old Dominion University was the institution with the strongest centrality, reaching 0.29. The research institution formed a core partner in 2017 with a scale of 14. The highest ranked by Sigma (∑) was the University of Massachusetts, reaching 1.58.

**Table 1:** Top 10 institution published analysis (2003–2022).

**Rank**	**Institution**	**Freq**	**Centrality**	**Sigma (∑)**
1	University of Delaware	21	0.18	1.00
2	University of Toronto	21	0.18	1.00
3	University of Massachusetts	14	0.15	1.58
4	Old Dominion University	14	0.29	1.00
5	University of Florida	12	0.05	1.00
6	Kansas State University	12	0.01	1.00
7	Oregon State University	12	0.01	1.00
8	Tufts University	11	0.05	1.00
9	Deakin University	10	0.05	1.00
10	Univ Wisconsin	9	0.01	1.00

Overall, the network size of the PA intervention in ASD research is small, the connection density is low, and most of the cooperation is mainly intra-regional cooperation. Consider, for example, the University of Delaware, the University of Florida, the University of Virginia, McMaster University, Oregon State University, and Texas Woman's University, there is a high density of intra-regional cooperation between universities in the United States. Similarly, the Chinese University of Hong Kong, the Education University of Hong Kong, Shenzhen University, and National Cheng Kung University in China have a high density of intra-regional cooperation at the core. After 2016, the overall density of the institutional cooperation network in the field of PA intervention increased, forming a large subnet, and the degree of cooperation was greatly improved, and cross-regional cooperation also appeared, such as a collaboration between the University of Toronto, Deakin University, University College London, McMaster University, and Yangzhou University. In addition, observing the overall high-frequency issuing agencies, Australia, North America, and Europe are more frequent, the remaining continents are small, and the cooperation density is also small. It can also be concluded that there is still a large space for cooperation between local institutions, and it is necessary to establish a more in-depth cooperative relationship of research institutions to promote the development of PA intervention in ASD research.

#### Co-author analysis

**Figure 4:**
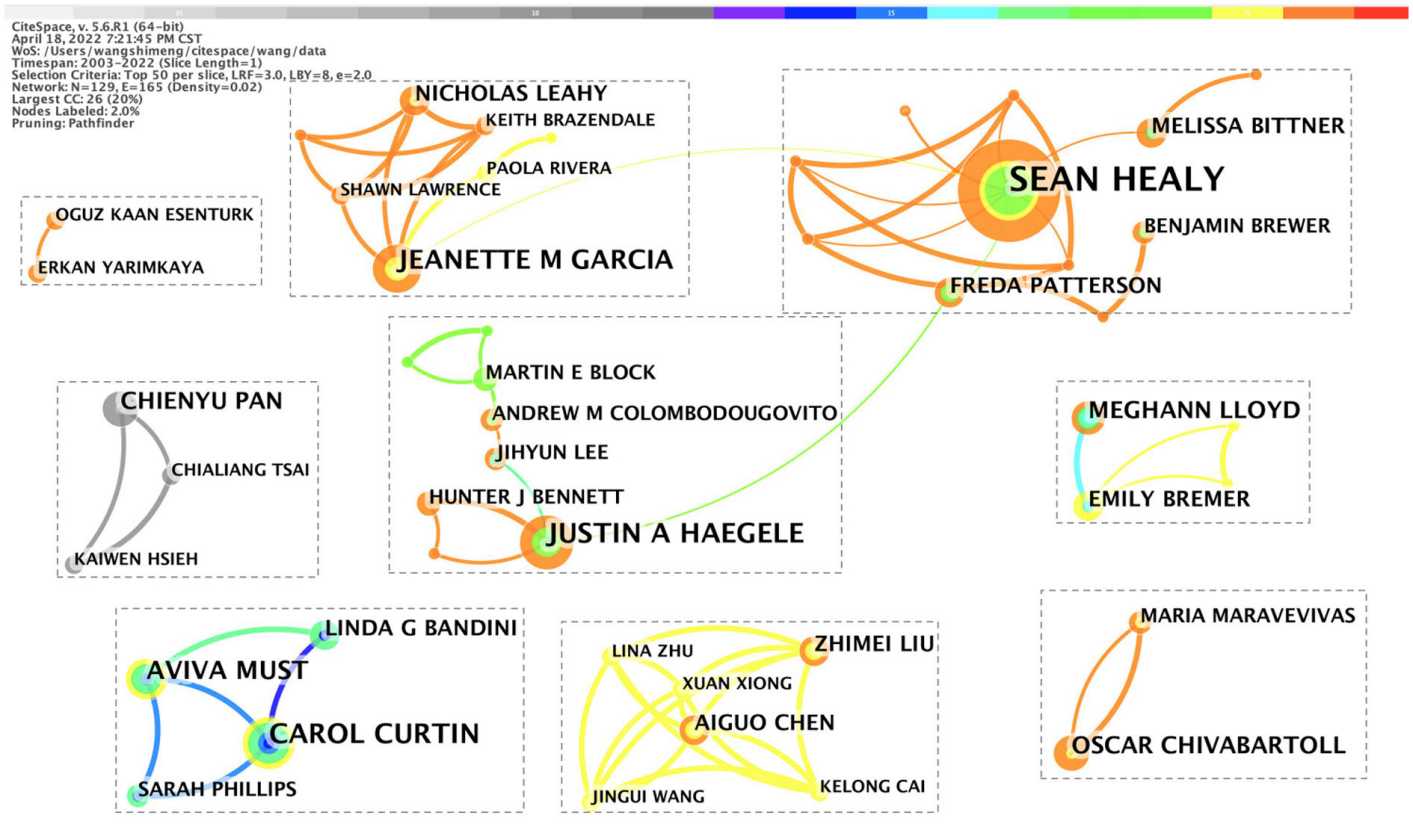
Co-authorship network (1 January 2003 to 1 March 2022). The color of the line represents the time the co-authors worked together. The larger the node diameter, the more studies the author has collaborated to publish. The thicker the line between the two authors.

By analyzing the author, the cooperative relationship with others could be investigated. We ran CiteSpace, generating networks as usual: 2003–2022; Slice length: 1 year; Select the node type: Author; Top N = 50; Selection Criteria Thresholding (c, cc): 1, 1; and Choice: Pathfinder and Pruning the merged network, and other parameter settings were likely to institutions. This study found knowledge mapping of the co-author with N = 129 and E = 165 (density = 0.02). Figure 4 shows the high density of author cooperation in the field of PA intervention in ASD, such as the cooperation network centered on Sean Healy, Carol Curtin, Justin A Haegele, Jeanette M Garcia, Óscar Chiva-Bartoll, Chieyu Pan, and Aiguo Chen.

Figure 4 and Table 2 show that Sean Healy's issued quantity in this field, was the highest, reaching 3.62, and an H-index was 17. His Research Topics mainly focus on increasing PA among youth with ASD and improving the motor skills of youth with developmental disabilities. The H-index of the second Carol Curtin reached 29, and it cited a total of 1,052 times; the research area is the prevalence of obesity in disabled populations, weight loss and PA interventions for adolescents with ID and autism, and observational studies on PA, dietary patterns, and/or obesity in children with various developmental disabilities. Justin A Haegele ranked third with an H-index of 28 and 3,054 citations on the interdisciplinary field of adapted PA, with a primary interest in examining how individuals with disabilities, more specifically those with visual impairments or ASD, experience PA participation. Jeanette M Garcia, the fourth most published author, had an H-index of 18 and 1,442 citations. Research fields mainly focus on three themes, namely, developing interventions to promote healthy behaviors in youth with ASD, measurement of PA and sleep quality in underserved populations, and community-based participatory research. Óscar Chiva-Bartoll, the fifth most published author, had an H-index of 20 and citations of 1,515. His research focuses on pedagogy and philosophy of sports and methodological innovation in physics Education. All of the above scholars have made great contributions to the area of PA intervention in ASD.

**Table 2:** Co-authorship and researcher's academic information (2003–2022).

**No**.	**Author**	**Freq**	**Burst**	**H-Index**	**Sum of cited**	**Research area**
1	Sean Healy	17	3.62	17	1,052	Disability, autism spectrum disorder, health behaviors, physical activity, 24-h activity cycle, mixed-methods research
2	Carol Curtin	9	3.22	29	5,202	Developmental disabilities, ADHD, autism, nutrition, health promotion
3	Justin A Haegele	9	0	28	3,054	Adapted physical education, adapted physical, activity, disability, autism spectrum disorder, diversity and inclusion
4	Jeanette M Garcia	8	0	18	1,442	Autism spectrum disorder, physical activity, social/emotional/behavioral disorders
5	Óscar Chiva-Bartoll	6	0	20	1,515	Physical education, transformative pedagogy, service-learning, and teacher education

*Burst refers to the specific time during which a sudden change in frequency occurs*.

*Sigma measures a combination of structural and temporal characteristics of nodes*.

*Data of H-Index, Sum of Cited, Research Area from Google Scholar*.

### Keyword analysis

#### Keyword co-occurrence analysis

The higher the frequency of the occurrence of keywords, the higher the probability of the keyword appearing in the research field, then the direction involved in the keyword may be more concerned by scholars, and the more likely it is that it is a hot issue in research. Table 3 shows that keyword frequency analysis helps clarify the research trends on PA intervention in ASD. PA and ASD were relatively high with frequencies of more than 350 times, because the above keywords are one of the important search terms for data sources, and they appear most frequently.

**Table 3:** Subjects of keyword co-occurrence analysis (2003–2022).

**No**.	**Keyword**	**Freq**	**Centrality**	**No**.	**Keyword**	**Freq**	**Centrality**
1	Autism spectrum disorder	398	0.09	11	Young Children	72	0.00
2	Physical activity	354	0.05	12	Disability	65	0.33
3	Children	312	0.22	13	Health	44	0.20
4	Adolescent	290	0.10	14	Prevalence	35	0.21
5	Autism	229	0.11	15	Youth	22	0.07
6	Exercise	155	0.10	16	Motor Skill	16	0.39
7	Intervention	114	0.13	17	Adult	15	0.00
8	Spectrum disorder	113	0.09	18	Individual	13	0.22
9	Obesity	101	0.02	19	Participation	13	0.22
10	Behavior	83	0.02	20	Asperger syndrome	7	0.12

Combined with keyword frequency, children, adolescents, exercise, intervention, and obesity were relatively high with frequencies over 100 times. It shows that these keywords are more concerned by scholars in this field, reflecting that the research direction related to these keywords is the core research content of the PA intervention in ASD research field, and the study finds that PA interventions and PA in children and adolescents with ASD improve symptoms such as stereotyped behavior and motor function in patients with ASD, as well as reducing childhood obesity rates and improving quality of life may be an important research direction in this field. In addition, inconsistencies in centrality and frequency were also found in this study, such as motor skill, prevalence, individual, participant disability, and other keywords with higher centrality but lower frequency. It reflects that although these research contents have an important bridging role in the research hotspots in this field, the degree of attention needs to be strengthened.

#### Keyword cluster analysis

**Figure 5:**
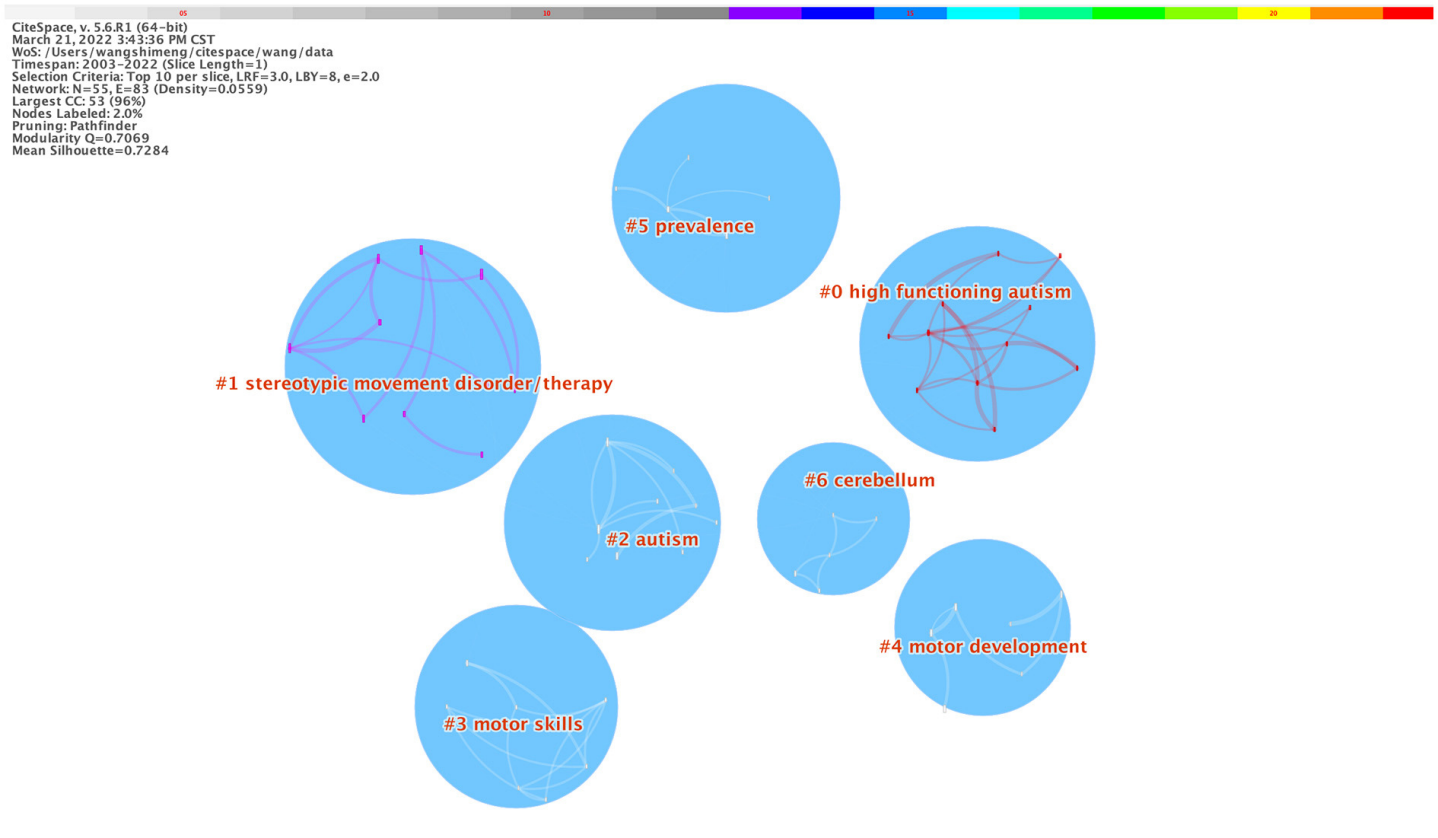
A landscape view of keyword cluster analysis generated by Top N = 10 per slice from 1 January 2003 to 1 March 2022.

The CiteSpace generated a network as usual: 2003–2022; Slice length: 1 year; Select the node type: Keyword; Top N = 10; and Pruning choice: Pathfinder, Pruning sliced networks, and Pruning the merged network. Given the co-occurrence of keywords, the nodes were revised, and the log-likelihood (LLR) algorithm was adopted for clustering calculation. The visualization map obtained in which N = 55 and E = 83 (density = 0.0559), the Modularity Q score was 0.7069, and the Mean Silhouette score was 0.7284, as presented in Figure 5. There was a total of 7 clusters (e.g., #0 high functioning autism, #1 stereotypic movement disorder/therapy, #2 autism, #3 motor skill, #4 motor development, #5 prevalence, and #6 cerebellum), as shown in Figure 5 and Table 4.

**Table 4:** Subjects of cluster analysis (2003–2022).

**Clusters**		**Silhouette**	**Size**	**Log-likelihood (LLR)**
#0	High functioning autism	0.926	11	Antecedent exercise, mouse model, gene, high functioning autism, computer science, program, individual, physical exercise, skill, activity pattern, mutation
#1	Stereotypic movement disorder/therapy	0.921	10	Student, youth, exercise, physical activity, stereotypic behavior, adolescent, autism spectrum disorder, health, adult, participation
#2	Autism	1	9	Stress, acquisition, meta, system, social behavior, children, asperger syndrome, spectrum disorder
#3	Motor Skills	0.95	7	Physical activity, meta-analysis, postural control, motor skill, intellectual disability, barrier, developmental disability
#4	Motor development	0.956	4	Behavior, environment, age, disability, intervention, young children
#5	Prevalence	0.858	5	Prevalence, psychiatric disorder, obesity, overweight, spectrum
#6	Cerebellum	0.944	5	Efficacy, asd, academic achievement, fitness, inhibitory control

Clusters #0 high functioning autism and #2 autism: It is a high-functioning ASD group, as a class of disorders with higher IQ and function in ASD and milder autism symptoms; because it has obvious PA intervention effect, it is easier to integrate into the general population. It has become the preferred target for most researchers to carry out autism PA interventions (Kosari et al., 2012; Keyhani and Kosari, 2015; Ferreira et al., 2018). All patients with Asperger's syndrome or high-functioning ASD were subjected to social skills or problem behaviors, and the results were remarkable. In addition, mouse models, m gene, and meta as one of the keywords of #0 and #2 clustering have attracted the attention of the academic community in recent years. ASD is associated with genetic factors for the first time by Korvatska et al. (2002). PLD2, PLD5, PCDH10, CDH8, MET, and CNTN3 play a role in axon growth. SHANK3 and CNTNAP2 (De Rubeis et al., 2014; Otazu et al., 2021) are also involved in synaptic development. These genes are in cell adhesion, ubiquitination and GTPase/RAS signal, and other pathways. Cell adhesion pathways will affect axon guidance and synapse formation. The ubiquitin pathway affects dendritic spines and postsynaptic dense matter development. Some important abnormal gene expressions cause changes in the related pathways that affected neuronal development, structure, and function, furthermore, affecting the cortical pattern of neural circuitry may ultimately lead to the occurrence of ASD (Holt and Monaco, 2011). Therefore, based on the behavioral detection method of mouse models, scholars have conducted a series of tests on the intervention of PA in patients with ASD, including detecting learning and social interaction. Stereotypes, memory, and anxiety, provide more scientific evidence for the effect of PA intervention on ASD. Developments in the field of research must be accompanied by scientific and objective evidence, which relies on a large number of high-quality randomized controlled trials as well as meta-analyses of randomized controlled trials. Therefore, research methods, such as Meta, are also hotspots in the current research field of PA intervention in ASD, which provide important tools for accurate and efficient analysis of massive data and verification of the validity of intervention prescriptions.

Cluster #1 stereotypic movement disorder/therapy: Stereotyped behavior is a major feature of patients with ASD in all groups, manifested as aimless repetition of single actions, which seriously affects the acquisition of functional behaviors and social skills in patients with ASD, and easily leads to self-harm, emotional, and other problems. Interventions in stereotyped behavior are significant for the ASD research field. In this regard, scholars have conducted a series of studies on the effect of PA intervention on stereotyped behavior in patients with ASD based on brain imaging technology (Sorensen and Zarrett, 2014). Numerous studies have shown that PA has become an effective means of improving stereotyped behavior in patients with ASD, such as the study of Minoei et al. (2015) to intervene in ASD through horseback riding. Significant improvements have been observed in stereotyped behavior in children with ASD. The effectiveness of PA interventions on children with ASD has also demonstrated in the meta-analysis of Teh et al. (2021), arguing that exercise interventions are an evidence-based and sustainable way to improve stereotyped behavior in children with autism. Therefore, both the initial exploration of autism exercise intervention 40 years ago and the study using a more rigorous experimental design today have demonstrated the positive effect of physical exercise in improving repetitive stereotyped behaviors in children with autism, which suggests that moderate and large-intensity exercise may be better.

Clusters #3 motor skill, #4 motor development, and #5 prevalence: The development of motor function in patients with ASD is highly correlated with their language, cognitive, and social development abilities. Studies have found that regardless of the IQ level of patients with ASD, there are a variety of motor function defects, including fine movements, coarse movements, postural control, imitation, or operation. Motor deficits limit social activity, affect the development of language, behavioral, and cognitive function in people with ASD, and increase ASD with long-term decreased levels of PA risk rates for overweight and obesity in patients (Healy et al., 2018). Scholars examined the evidence of the effectiveness of PA interventions in children with autism and found that increasing PA in children and adolescents with ASD improved body mass index (BMI) and physical health, effectively alleviating their obesity rates and prevalence of ASD (Craig et al., 2021). Therefore, it can be learned that the main research content of the current research field of PA intervention on ASD motor function is oriented to the development of the motor function, reducing the risk rate of all diseases caused by motor function defects. Scientific evidence is added to the study of PA intervention in the auricular function of ASD.

Cluster #6 cerebellum: From the perspective of the role of PA in promoting the development of the cerebellum in individuals with ASD, studies have shown that individuals need the participation of the cerebellum in the process of motor control and movement; in addition, the cerebellum can also help individuals obtain and identify sensory information and has the function of coordinating muscle movement and maintaining balance in the body (Desmond and Fiez, 1998). Despite the above functions of the cerebellum, unfortunately, individuals with ASD have defects in cerebellar tissue. For example, in 95% of individuals with ASD, the cerebellum develops malformations (Abu-Elneel et al., 2008). In addition, there was developmental insufficiency in the posterior cerebellar worms and hemispheres in individuals as well as loss of Purkinje and granulocytes (Fatemi et al., 2012). Based on observations of individuals with ASDs, combined with an analysis of cerebellar function, it can be seen that cerebellar abnormalities may be a factor explaining the causes of autism. It is well-known that most exercise programs require the cortex involvement of vision, hearing, and proprioception, all of which are related to the function of the cerebellum. Therefore, it might be considered that the PA intervention is precise because the cerebellum of the individual with ASD is fully stimulated and developed so that the level of motor skills can be improved, improving their social communication skills. Clinical practice confirms that exercise promotes cerebellar neurogenesis, improves cerebellar mitochondrial function, reduces oxidative stress (Marques-Aleixo et al., 2015), and enhances the anti-apoptotic effect of neurons in the cerebellum (Seo et al., 2013).

#### Keyword burst analysis

To obtain the research frontier and development trend of PA intervention on ASD, the strongest keywords cited in the literature were analyzed. Keyword burst refers to keywords appearing suddenly in a short period or which usage frequency increases sharply. Overall, it reveals the evolution of the Research Topic in different periods. In this study, 7 keywords highlighted by citations are obtained through analysis, as listed in Table 5.

**Table 5:** Seven keywords with the strongest citation bursts (2003–2022).

**Keywords**	**Year**	**Strength**	**Begin**	**End**	**2003-2022**																			
Skill	2003	4.1269	2008	2012	**—**	**—**	**—**	**—**	**—**	**—**	**—**	**—**	**—**	**—**	**—**	**—**	**—**	**—**	**—**	**—**	**—**	**—**	**—**	**—**
Youth	2003	4.4705	2008	2014	**—**	**—**	**—**	**—**	**—**	**—**	**—**	**—**	**—**	**—**	**—**	**—**	**—**	**—**	**—**	**—**	**—**	**—**	**—**	**—**
Participation	2003	6.7935	2010	2015	**—**	**—**	**—**	**—**	**—**	**—**	**—**	**—**	**—**	**—**	**—**	**—**	**—**	**—**	**—**	**—**	**—**	**—**	**—**	**—**
Asperger syndrome	2003	4.4041	2010	2012	**—**	**—**	**—**	**—**	**—**	**—**	**—**	**—**	**—**	**—**	**—**	**—**	**—**	**—**	**—**	**—**	**—**	**—**	**—**	**—**
Prevalence	2003	4.2786	2013	2016	**—**	**—**	**—**	**—**	**—**	**—**	**—**	**—**	**—**	**—**	**—**	**—**	**—**	**—**	**—**	**—**	**—**	**—**	**—**	**—**
meta	2003	10.9935	2017	2020	**—**	**—**	**—**	**—**	**—**	**—**	**—**	**—**	**—**	**—**	**—**	**—**	**—**	**—**	**—**	**—**	**—**	**—**	**—**	**—**
Sedentary behavior	2003	3.3561	2020	2022	**—**	**—**	**—**	**—**	**—**	**—**	**—**	**—**	**—**	**—**	**—**	**—**	**—**	**—**	**—**	**—**	**—**	**—**	**—**	**—**

–*: shows which period the citation burst is the strongest. For instance, the Skill has the longest period of burst from 2008 to 2012*.

According to Table 5, no hot keywords have been identified in the field of research on PA interventions for ASD during the period of 2003–2008. Since 2008, skill and teen keywords have received widespread attention from scholars. Skill is an important object of PA intervention for ASD such as social skills and motor skills. The study found that overall motor skill scores were significantly lower in children with ASD compared to developing normal children, and social skills scores in children with severe movement impairment were significantly lower than in mild children (Green et al., 2009). Pan (2011) demonstrated in an experimental study that PA has a certain role in promoting water sports skills in children with ASD. Healy et al. (2018) also showed in a meta-analysis that PA has a positive impact on proactive skills, locomotor skills, and skill-related fitness. Since 2010, the key words Participation and Asperger syndrome have attracted wide attention from scholars. Asperger's syndrome is one of the syndromes of ASD, but its symptoms are mild compared to autism. Patients with Asperger's syndrome have better intelligence and language expression than those with autism but have some difficulties in social interaction. Borremans et al. (2010) compared the physical fitness and PA of ordinary adolescents with Asperger's syndrome and found balance, coordination, and coordination among adolescents with Asperger's syndrome. Abilities such as flexibility are lower than those of normal adolescents. As the intervention effect of Asperger's syndrome subjects is large, it has become the preferred object for most researchers to carry out autism PA intervention and has also become a research hotspot. Between 2013 and 2016, prevalence became a research hotspot in the field of PA interventions in ASD, and to this day, according to the Centers for Disease Control and Prevention, the prevalence of ASD has increased from 2 in 10,000 to 1 in 54 over the past 20 years (Maenner et al., 2020). Not only the prevalence of ASD but also the rate of obesity due to ASD symptoms has continued to rise. Studies have found that PA has an improving effect on the motor skills of ASD, which greatly reduces the obesity rate of patients with ASD. Over the past decade, experimental studies on PA interventions for ASD have gradually increased; however, developments in the field of research must obtain scientific and objective evidence, which relies on a large number of high-quality randomized controlled trials as well as meta-analyses of randomized controlled trials. Therefore, for the period of 2017–2020, research methods such as systematic review and meta-analysis are also hotspots in the field of PA intervention in ASD research, which also indicates that the research methods in this research field are accurate and efficient analysis of massive data. Validating the effectiveness of intervention prescriptions provides an important tool. In addition, inconsistencies in centrality and frequency were also found in this study, such as *de novo* syndrome, intellectual disability, and other keywords with higher centrality but lower frequency. It reflects that although these research contents have an important bridging role in the research hotspots in this field, the degree of attention needs to be strengthened. During 2020 to 2022, sedentary behavior became a hot keyword. With the development of the society, the application of electronic products is more and more widely, greatly influenced people's life. The popularity of electronic products reduces the time of physical activity of children with ASD, and increases the sedentary behavior of children with ASD. Studies have shown that PA can significantly improve sedentary behavior in children with ASD (Thompson et al., 2022). Therefore, the relationship between ASD children and Screen Time may be a new trend in future research on sedentary behavior.

## Conclusion

This study systematically analyzed the research literature in the field of PA intervention in ASD from 1 January 2003 to 1 March 2022, using bibliometric analysis and visual analysis. The results are summarized as follows. First, the literature on PA interventions focused on ASD research shows a growing trend. It is speculated that the literature in this field will continue to grow for some time in future; in this field, the leading institution in this field is the University of Delaware; many authors have formed a network of collaborators in the field, such as Sean Healy and Carol Curtin. Second, the focus of this research area mainly includes PA interventions for children and adolescents with ASD and PA to improve symptoms such as stereotyped behaviors and motor function in patients with ASD as well as to reduce childhood obesity rates and improve quality of life of PA intervention in ASD. Third, the hotspot analysis of PA intervention in ASD research found skill, youth, prevalence, and meta-analysis systematic reviews. It is the long-term concern and focus of researchers.

In summary, as the current research status is only of short term, it is not possible to verify the long-term effect. It is a future trend to explore the long-term effects of PA interventions on ASD and to bring the best rehabilitation means to patients with ASD. Moreover, with the rapid development and mature application of brain imaging technology, brain imaging technology has become an important means to explore the underlying neural mechanisms of ASD psychology and behavior and to reveal the structure and functional characteristics of the human brain. Using brain imaging technology to analyze the mechanism of PA intervention for ASD, conducting more studies to replicate and expand the existing findings, testing the sustainability of these benefits for children with autism, and constructing a multidimensional exercise integrated intervention model are the main directions of future research in this field.

## Limitation

In this study, the CiteSpace software was used to analyze current studies on PA intervention in ASD. It provides some thinking for scholars in the field of ASD to have a comprehensive understanding of the characteristics, hotspots, and development trends of the current research field and to explore intervention methods. However, there are some limitations to this study. For example, CiteSpace cannot process documents in multiple databases at the same time, and only the documents in the WoS database can be processed. Moreover, the literature of research and analysis in the English language may cause the conclusion to service because of a lack of relevant literature materials. Therefore, future research should consider analyzing a variety of databases, and multiple languages, to increase research conclusions.

## Data availability statement

The original contributions presented in the study are included in the article/Supplementary material, further inquiries can be directed to the corresponding author/s.

## Author contributions

AC and SW: conceptualization. SW and DC: methodology, writing—original draft preparation, investigation, data curation, and visualization. SK, AC, and IY: writing—review and editing. AC: funding acquisition. All authors have read and agreed to the published version of the manuscript.

## Funding

This research was funded by the National Natural Science Foundation of China [Grant number 31771243].

## Conflict of interest

The authors declare that the research was conducted in the absence of any commercial or financial relationships that could be construed as a potential conflict of interest.

## Publisher's note

All claims expressed in this article are solely those of the authors and do not necessarily represent those of their affiliated organizations, or those of the publisher, the editors and the reviewers. Any product that may be evaluated in this article, or claim that may be made by its manufacturer, is not guaranteed or endorsed by the publisher.
